# *Ridleyandra merohmerea* (Gesneriaceae), a new species from Kelantan, Peninsular Malaysia

**DOI:** 10.3897/phytokeys.89.20344

**Published:** 2017-10-17

**Authors:** Siti-Munirah Mat Yunoh, Zaharil Dzulkafly

**Affiliations:** 1 Forest Research Institute Malaysia, 52109 Kepong, Selangor, Malaysia; 2 118, Jalan Puncak Jelapang 2, Puncak Jelapang Maju 30020 Ipoh, Perak, Malaysia

**Keywords:** Conservation status, endemic, flora, Gesneriaceae, lowland dipterocarp forest, Malaysia, *Ridleyandra*

## Abstract

*Ridleyandra merohmerea*, a new species of Gesneriaceae, is described and illustrated. It is endemic in Peninsular Malaysia and known from a few populations along the Tuang River in the lowland dipterocarp forest of the Ulu Galas Forest Reserve in Kelantan, Peninsular Malaysia. Its conservation status is assessed as Critically Endangered.

## Introduction

*Ridleyandra* is a genus of Gesneriaceae with 31 species distributed from Thailand, Peninsular Malaysia and Borneo (Weber and Burtt 1998, Kiew 2009, 2011, 2013, 2014, Siti-Munirah 2012). In Peninsular Malaysia, 23 species are recognized and all of them are endemic. This new species, *Ridleyandra merohmerea* was first noticed and discovered by second author, Mr Zaharil Dzulkafly, a professional nature photographer, in January 2017 with a small population growing along the riverbank of Sungai Tuang. As a result of further examination at the site by the authors, this species appears to be new to science for its notable striking large trumpet-shaped flowers with bright red corolla tube and much darker red lobes and inner surface of the tube that are almost black.

## Taxonomy

### Ridleyandra merohmerea

Taxon classificationPlantaeLamialesGesneriaceae

M.Y.Siti-Munirah & D.Zaharil sp. nov.

urn:lsid:ipni.org:names:60475282-2

Figure 2 , 3

#### Diagnosis.

In its leaf shape and flower lobe colour, *Ridleyandra merohmerea* most resembles *R. iminii* Siti-Munirah but it differs in its leaves that are flat above and not wavy (glossy above and wavy), petiole 1–2 cm long (1–4 cm), oblanceolate and falcate lamina (lamina lanceolate oblong and not falcate), peduncle 8–13 cm long with green to dark purple (5–8 cm, pale green), pedicels 1–2 cm long (2.5–3 cm). In flower colour also is similar to *R. iminii* but differs in the sepal dark purple outer surface (sepals light green), corolla tube completely bright red outside (fully white outside).

#### Type.

MALAYSIA. Peninsular Malaysia: Kelantan, Ulu Galas Forest Reserve, Sungai Tuang, 5 April 2017, (fl & fr) Siti-Munirah FRI 76345 (holotype: KEP!).

#### Description.

Perennial herb. **Stem** woody, unbranched, erect, 2–30 cm tall, 3–6 mm diam., upper part of stem, petiole, lower surface of veins with greenish white-brown, dense, unbranched, multiseriate, hairs ca. 1–3 mm long. **Leaves** in unequal pairs, clustered in a rosette at the top of the stem, petioles 1–2 cm long; lamina oblanceolate, falcate, 6–20 × 2–5 cm, flat above, narrowed to base, margin lobed, tooth tip acute or rounded, 2–15 × 1.5–10 mm, apex acute to attenuate, green above, whitish green beneath; midrib impressed above, prominent beneath, lateral veins 10–17 pairs. **Inflorescence** single-flowered, peduncle 8–13 cm long, green to dark purple, slightly curved downward; bracts in pairs, opposite or alternate, pale green, lanceolate, ca. 3 × 1.5 mm, pedicel 1–2 cm long, dark purple red, hairs brown. **Flowers** with sepals 5, oblanceolate, ca. 3 × 1.5 mm, dark purple outside, green inside; corolla trumpet-shaped, tube ca. 5 cm long, ca. 1.5 cm diam. at the mouth, dilating to ca. 0.5 cm at the base, lobes ca. 7–9 mm × 5–6 mm, outside minutely pubescent, white at the base becoming completely bright red outside, inside completely deep red, lobes dark red to almost black projecting ca. 9 mm beyond the tube, lateral lobes ca. 7 × 6 mm and the centre lobe ca. 9 × 5 mm; stamens 4 in 2 pairs, filaments white to maroon, lower pair ca. 3 cm long, upper pair ca. 3.5 cm long, anthers creamy yellow, ca. 1 mm long, joined in pairs; nectary annular, ca. 1 mm high; ovary ca. 4 cm long, pale mauve, stigma purple, broadly spathulate, ca. 2 mm × 1–2 mm. **Capsules** dark purple or greenish, slightly curved downward, glabrous, 6–6.5 cm long, 3–6 mm diam.

#### Distribution.

**Map 1.**
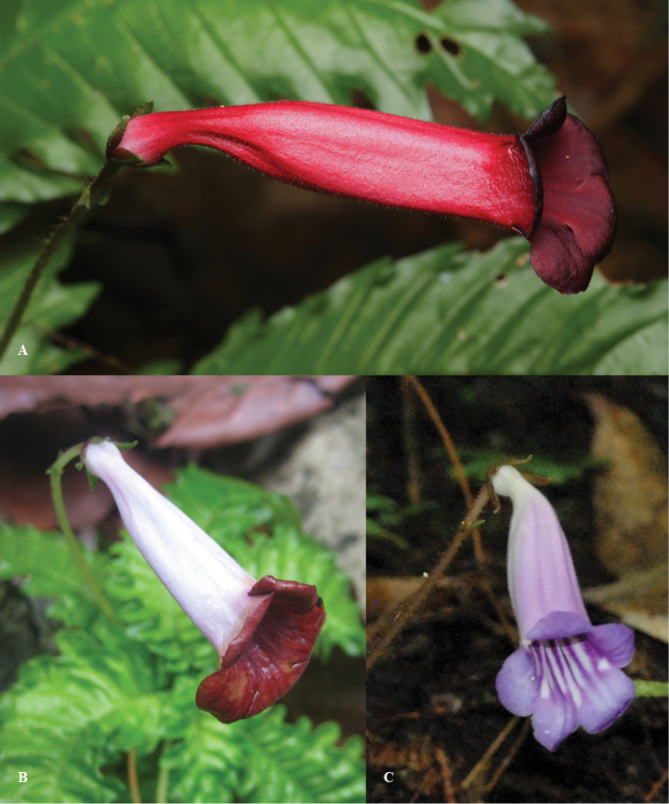
Distribution of *Ridleyandra merohmerea* (●), *R. morganii* (▲) and *R. iminii* (■) in Peninsular Malaysia. NFI III Courtesy of Forest Department Peninsular Malaysia.

Endemic in Peninsular Malaysia, Kelantan, Gua Musang (Ulu Galas Forest Reserve, Sungai Tuang (Map 1). 4°42.425'N, 101°55.831'E.

#### Ecology.

**Figure 1.**
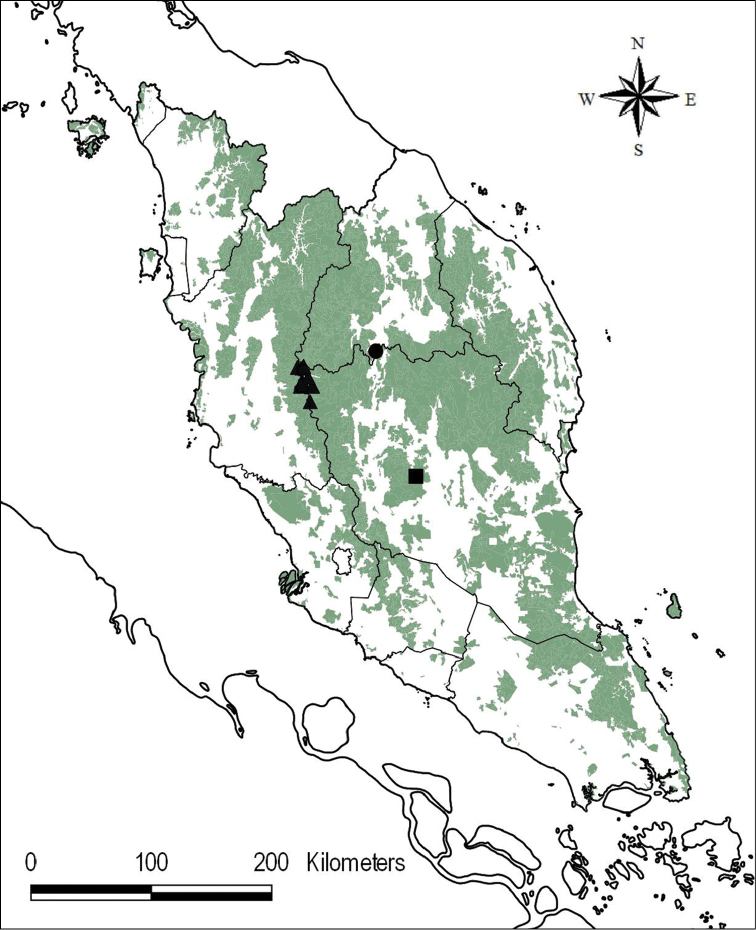
Habitat of *Ridleyandra merohmerea*. **A** habitat beside the river (plant show by red arrow) **B** plant habit **C** from left; Zaharil D, Mohd Hairul MA, Wan Syafik WP & Siti-Munirah MY; beside the habitat of *Ridleyandra merohmerea* **D–F** occurs on steep slope and some on rocks just beside the river (Photo by **A–B, D–F** Siti-Munirah MY **C** Zaharil D).

Occurs on the river bank, steep slope and some on rocks just beside the river (Figure 1), mostly in under shaded conditions. All populations occurred at about 260-300 m above sea level (asl).

**Figure 2.**
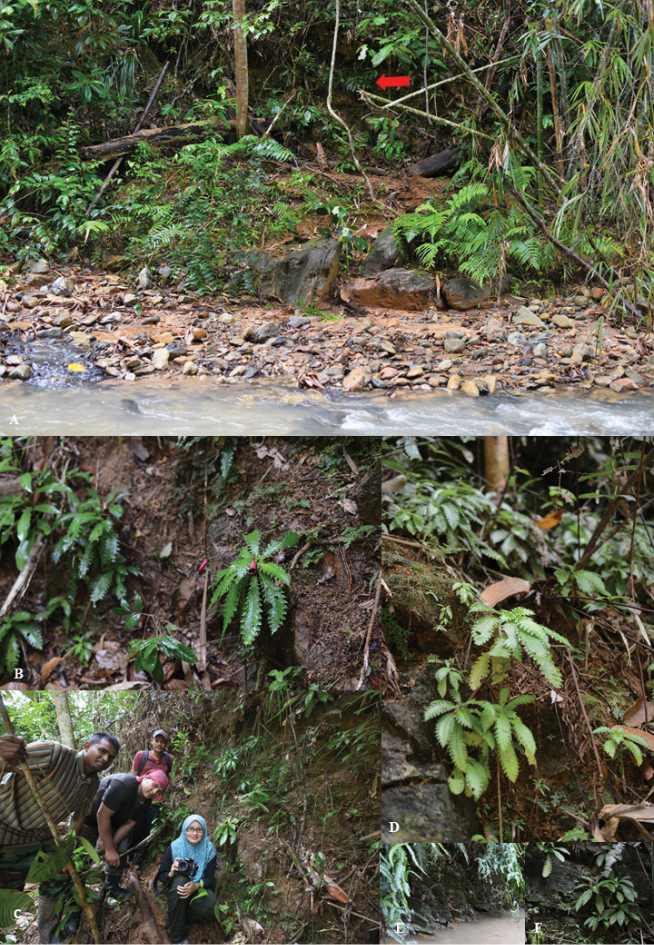
*Ridleyandra merohmerea* Siti Munirah & Zaharil. **A, B** habit **C** leaf arrangement **D** petiole with hairs **E** flower from side view **F** peduncle with hairs **G** corolla curve **H** bracts **I** sepals **J** nectary **K** flower with five lobes **L** flower lobes dark red **M** corolla outer surface **N** corolla surface inside with stamens **O** pistil **P** stigma **Q** anthers **R** fruit capsule open **S** young fruit (Photo: **A**–**K, M–S** Siti-Munirah MY, **L** Zaharil D).

**Figure 3.**
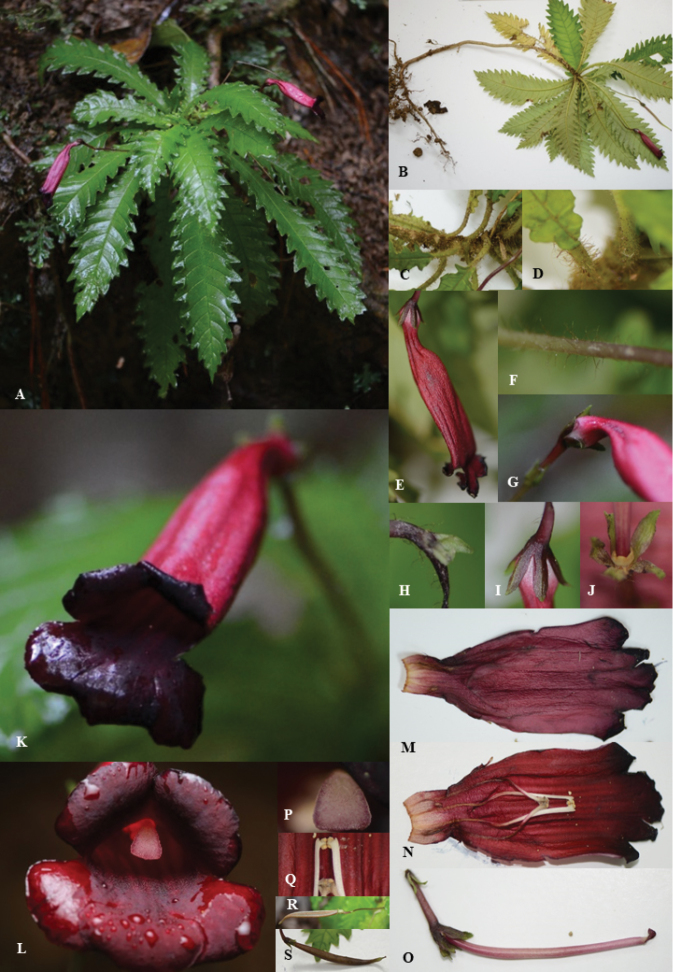
*Ridleyandra merohmerea* Siti Munirah & Zaharil. **A** habit with flower and fruit **B** flower **C** corolla (front view) **D** calyx (sepals) **E** bract **F** corolla opened to show position of stamen **G** anthers (upper pair) **H** anthers (lower pair) **I** pistil All from Siti Munirah FRI 76345. DRAWN BY M. AIDIL.

#### Etymology.

From the local dialect of Kelantanese people, ‘merohmerea’ means ‘merah terang’ in Malay and bright red in English. This name was chosen in the hope it will attract more Kelantanese to be more interested, concerned and aware of the importance of forest biodiversity in Kelantan especially since recently there are so many controversies and issues about disturbance to forests in Kelantan that in some cases might result in destruction of this new unique discovery. Hopefully, this name will attract more stakeholders to understand the importance of protecting and conserving the forest and this beautiful species and to realise that yet more new species are waiting to be discovered.

#### Conservation status.

CR B2ab(ii,iii). Following the 2012 IUCN Red List Categories and Criteria, (IUCN 2012) this species is assessed as critically endangered because it is only known from only one locality. Furthermore, only about 150 individuals were counted scattered along the river from the three populations found, currently it is being threatened all three populations are exposed by the rubber tree plantation project, which is currently ongoing within the forest reserve (or compartments). Although the three *Ridleyandra* populations lie within the Ulu Galas Forest Reserve, unfortunately the status of the forest is uncertain because the area had been approved for timber latex clone (TLC) rubber tree plantation and will soon be converted to plantation (‘Projek Ladang Hutan’). None of the populations in Ulu Galas FR is in a Totally Protected Area.

#### Note.

**Figure 4.**
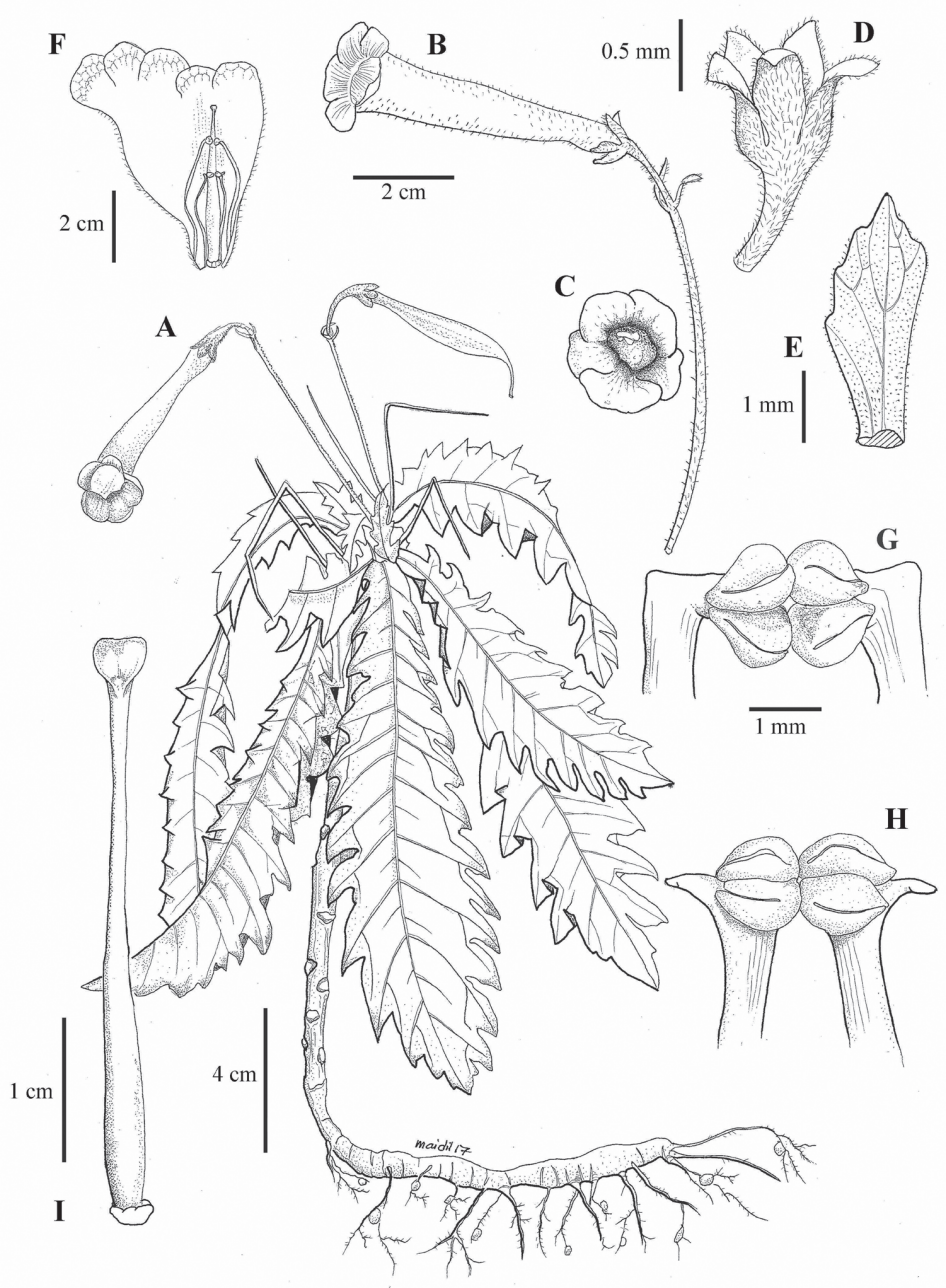
Comparison of resembles species **A** *Ridleyandra merohmerea* **B** *Ridleyandra iminii* **C** *Ridleyandra morganii* (Photo by **A** Zaharil D **B**–**C** Siti-Munirah MY).

**Figure 5.**
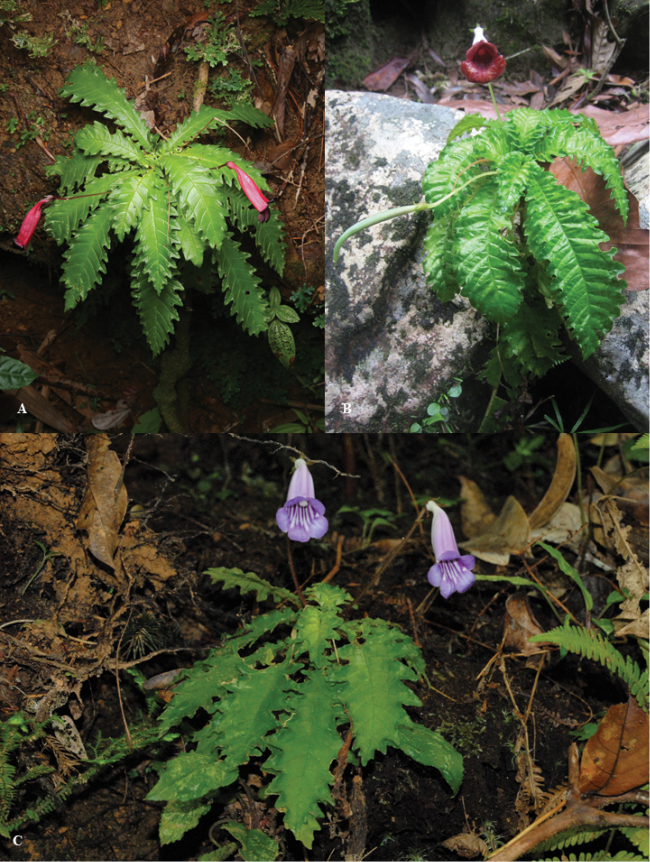
Comparison flowers of resembles species **A** *Ridleyandra merohmerea* **B** *Ridleyandra iminii* **C** *Ridleyandra morganii* (Photo by **A** Zaharil D **B**–**C** Siti-Munirah MY).

Based on lobed margin leafs, *R. merohmerea* also close to *R. morganii* (Franch.) A. Weber, however, in other character, including the inflorescence it is totally different (Figure 4, Table 1). In addition, phytogeography also different when referring to *R. iminii* Siti Munirah which endemic to Gunung Benom (Pahang) on upper hill dipterocarp forest, *R. merohmerea* endemic to Galas FR (Kelantan) in lowland dipterocarp forest, while *R. morganii* is quite wide distribute on central main range at many on montane forest such as Gunung Brinchang, Pahang (Map 1). *Ridleyandra merohmerea* is a distinctive species among *Ridleyandra* species in its flower color (Figure 5). Indeed, the only other *Ridleyandra* species with a red flower is *R. iminii*, however the corolla outer surface of *R. iminii* is white, while in *R. merohmerea* is completely red.

**Table 1.** Main morphological differences among the similar species of *Ridleyandra*.

Character	*R. merohmerea*	*R. iminii*	*R. morganii*
Leaves			
*petiole* (cm long)	1–2	1–4	0.5–2
*lamina* (surface)	flat not wavy	wavy	flat not wavy
Inflorescence			
*peduncle* (cm long)	8–13	5–8	7–10
*pedicel* (cm long)	1–2	2.5–3	0.5–0.8
Flowers			
*sepals* (shape)	oblanceolate	lanceolate	elliptic
*sepals* (colour)	dark purple to green	light green	purple
*corolla tube* (outside)	bright red	white	purple
*corolla tube* (inside)	deep red	dark red	purple with white lines
*lobes* (colour)	dark red to almost black	dark red	purple

## Supplementary Material

XML Treatment for Ridleyandra merohmerea
